# Telepsychiatry During the COVID-19 Pandemic: Development of a Protocol for Telemental Health Care

**DOI:** 10.3389/fpsyt.2020.552450

**Published:** 2020-09-23

**Authors:** Rodrigo Ramalho, Frances Adiukwu, Drita Gashi Bytyçi, Samer El Hayek, Jairo M. Gonzalez-Diaz, Amine Larnaout, Paolo Grandinetti, Marwa Nofal, Victor Pereira-Sanchez, Mariana Pinto da Costa, Ramdas Ransing, Andre Luiz Schuh Teixeira, Mohammadreza Shalbafan, Joan Soler-Vidal, Zulvia Syarif, Laura Orsolini

**Affiliations:** ^1^ Department of Social and Community Health, School of Population Health, University of Auckland, Auckland, New Zealand; ^2^ Department of Neuropsychiatry, University of Port Harcourt Teaching Hospital, Port Harcourt, Nigeria; ^3^ Mental Health Center Prizren, Hospital and University Clinical Service of Kosovo, Prizren, Kosovo; ^4^ Department of Psychiatry, American University of Beirut, Beirut, Lebanon; ^5^ CERSAME, School of Medicine and Health Sciences, Universidad del Rosario—Clínica Nuestra Señora de la Paz, Bogotá, Colombia; ^6^ Razi Hospital, Faculty of Medicine of Tunis, Tunis El Manar University, Tunis, Tunisia; ^7^ Addictions Services (SerD), Department of Territorial Assistance, ASL Teramo, Teramo, Italy; ^8^ Helwan Mental Health Hospital, Cairo, Egypt; ^9^ Department of Child and Adolescent Psychiatry, NYU Grossman School of Medicine, New York, NY, United States; ^10^ Unit for Social and Community Psychiatry, WHO Collaborating Centre for Mental Health Services Development, Queen Mary University of London, London, United Kingdom; ^11^ Institute of Biomedical Sciences Abel Salazar, University of Porto, Porto, Portugal; ^12^ Hospital de Magalhães Lemos, Porto, Portugal; ^13^ Department of Psychiatry, BKL Walalwalkar Rural Medical College, Maharashtra, India; ^14^ Department of Childhood and Adolescent Psychiatry, Universidade Federal do Rio Grande do Sul, Porto Alegre, Brazil; ^15^ Mental Health Research Center, Iran University of Medical Sciences, Tehran, Iran; ^16^ Fidmag Research Foundation, Hermanas Hospitalarias, Barcelona, Spain; ^17^ Department of Psychiatry, Tarakan General Hospital, Jakarta, Indonesia; ^18^ Unit of Clinical Psychiatry, Department of Clinical Neurosciences/DIMSC, School of Medicine, Polytechnic University of Marche, Ancona, Italy

**Keywords:** ****COVID-19, Coronavirus disease, mental health, protocol, psychiatry, telemedicine, telemental health, telepsychiatry

## Abstract

**Background:**

The rapid spread of the Coronavirus disease 2019 (COVID-19) has forced most countries to take drastic public health measures, including the closure of most mental health outpatient services and some inpatient units. This has suddenly created the need to adapt and expand telepsychiatry care across the world. However, not all health care services might be ready to cope with this public health demand. The present study was set to create a practical and clinically useful protocol for telemental health care to be applied in the context of the current COVID-19 pandemic.

**Methods:**

A panel of psychiatrists from 15 different countries [covering all World Health Organization (WHO) regions] was convened. The panel used a combination of reactive Delphi technique and consensus development conference strategies to develop a protocol for the provision of telemental health care during the COVID-19 pandemic.

**Results:**

The proposed protocol describes a semi-structured initial assessment and a series of potential interventions matching mild, moderate, or high-intensity needs of target populations.

**Conclusions:**

Telemedicine has become a pivotal tool in the task of ensuring the continuous provision of mental health care for the population, and the outlined protocol can assist with this task. The strength of this protocol lies in its practicality, clinical usefulness, and wide transferability, resulting from the diversity of the consensus group that developed it. Developed by psychiatrists from around the globe, the proposed protocol may prove helpful for many clinical and cultural contexts, assisting mental health care providers worldwide.

## Introduction

The Coronavirus disease 2019 (COVID-19) pandemic has placed the world in an exceptional situation, forcing communities and governments to make fast decisions. The World Health Organization (WHO) has highlighted the importance of measures aimed at delaying the spread of the virus (1). Among these measures, there are non-pharmaceutical interventions, a critical part of current public health measures addressing the pandemic (2–4). These interventions aim at protecting people by physically distancing those with confirmed and suspected COVID-19 or potentially carrying the virus from the general population (2). Such interventions include physical distancing, quarantining, mandatory or voluntary isolation, closing national borders and other travel-related restrictions, closing schools and workplaces, and canceling social gathering events (2).

The rapid spread of the disease, along with the public health measures taken to reduce its progression, present a challenge to mental health services around the world, both in terms of a potential higher demand and difficulties in providing onsite services (5). In this scenario, telemedicine services provide a vital asset for mental health care (6). Telemedicine is defined as the use of telecommunication technologies to provide remote health care (7). The COVID-19 pandemic has created the need to expand telepsychiatry care; in fact, telepsychiatry use among mental health professionals has increased worldwide (8, 9). Unfortunately, these services are limited in various countries, and not all health care services around the globe might be ready to cope with this public health demand (10). Also, mental health care professionals and service providers may have feelings of apprehension or ill-preparedness when facing the sudden need to set up telepsychiatry services and/or to provide mental health care primarily *via* this approach (10). At the same time, not all services count with local guidelines for telepsychiatry (11), and available guidelines may not be transferable to different social and cultural contexts. Moreover, not all available guidelines may have contemplated the particular circumstances imposed by the current COVID-19 pandemic. The present project was set to assist with this situation, with the goal of creating a practical and clinically useful protocol for mental health care that would cater to most clinical and cultural contexts, assisting mental health care providers around the world.

## Methods

The project sought to take advantage of the knowledge and experience of psychiatrists from a wide range of countries, connected by the Early Career Psychiatrists Section of the World Psychiatric Association (12). A panel of 16 psychiatrists from 15 different countries covering all WHO regions was convened. The study used a combination of a modified Delphi technique, called reactive Delphi, and strategies drawn from the consensus development conference method (13–15) (**Tables 1**, **2**).

**Table 1:** Protocol development pathway.

Consensus group identified and invited to participate	
↓	
Set of trigger questions and topics to consensus group*	
↓	
1st draft developed incorporating replies	
↓	
1st draft to consensus group (reactive Delphi)	
↓	
2nd draft to consensus groups (consensus conference)	
↓	
Final draft	

*See **Table 2**.

**Table 2:** Trigger questions and topics.

* Each of the questions and topics prompted contributors to reply about what was applicable in their countries. Was telepsychiatry something already used before the pandemic? To what extent and in which way? How did the pandemic change that? Which is the most widely used tool (e.g., videoconferences, audio calls, text messages, instant messaging mobile apps, phone lines/call centers)? Are there any pre-consultation screenings? Who is handling the consultations conducted in this way (e.g., nurses, psychologists, psychiatrists)? What are people most commonly consulting for? Reception and acceptability by patients Technical and bureaucratic resources and challenges Are there any protocols or guides being used in your country? Level of training regarding telepsychiatry before and after the pandemic Role of early career psychiatrists in telepsychiatry Any suggestions on what should be something to consider when drafting recommendations?

All participants were provided with a set of trigger questions and topics *via* the social network messaging application WhatsApp^®^ and prompted to reply about what was applicable in their countries (**Table 2**). All answers were collected by the lead (RRam) and co-lead (LO) authors and entered into an Excel spreadsheet. Informed by these answers, RR developed a first draft of the protocol with the support of LO and then shared it with all participants as a Google^®^ document, who were asked to provide feedback using a reactive Delphi Technique (14, 15). All feedback was incorporated into a second draft and then shared and discussed with all participants using the Zoom^®^ platform and strategies drawn from the consensus development conference method (13). The final draft was unanimously accepted by all participants (**Table 1**). Group discussions were accompanied with a review of the emerging scientific literature about COVID-19 and its impact on mental health, as available in online journals and databases. Also, the answers provided to the trigger questions and topics informed the development of other manuscripts published (11) and to be published in the near future.

This study did not involve the management of sensitive data; all of the authors participated voluntarily, and their contributions reflect their own views and not necessarily those from their institutions. Due to the nature of this study, prior assessment by an Institutional Review Board was not necessary.

## Results

The consensus group was composed of 16 participants representing countries from all WHO regions: African Region, Region of the Americas, South-East Asia Region, European Region, Eastern Mediterranean Region, and the Western Pacific Region. These were representatives from lower middle income (Egypt, India, Indonesia, Nigeria, and Tunisia), upper middle income (Brazil, Colombia, Iran, Kosovo, and Lebanon), and high income countries (Italy, New Zealand, Portugal, Spain, and United States of America).

### Delivery Platform

There are some contexts where video conferencing is available for specialists and the population. However, while it would be ideal for everybody to have access to the tools and the necessary digital literacy to liaise with mental health providers online, this is not always the case. An actual means available for the wider population and mental health care providers in most contexts involve phone calls (landline or mobile), primarily *via* telephone hotlines. The following protocol was created with this limitation in mind. Still, whenever possible, video conferencing should also be made available to the public.

### Resources Required

We recommend mental health departments to provide an entry point to mental health care *via* telephone hotlines or helplines. But before making this service widely available, these departments should first organize the necessary resources, including human resources. This organization should include securing the contact information of, and/or an open line of communication with, available hospitals, ambulance services, and any other potentially necessary resource (e.g., hotlines for people experiencing intimate partner violence). Depending on availability, this organization should also include securing up-to-date information about COVID-19, public health measures, essential services, and any financial assistance available to the population in times of quarantine. It should also include setting up a filing system for records, if these were to be used.

The service would require, ideally, a coordination team, a technical support team, and mental health care providers (from here on referred to as providers). The coordination team should be responsible for ensuring resources, both material and human resources, including technical support. In case that not all members of the providers team were mental health specialists, the protocol we are presenting offers a guide for when to refer the call to these specialists. All providers should possess the appropriate and necessary competencies in terms of mental health care. In regard to the provision of telemental health care, it might be necessary to organize brief training at the service, either through professional associations, by inviting national or international consultants, or even through active learning under a self-training scheme. There may be contexts where it would be the same providers who are in charge of coordinating the service. Still, it is advisable for them to organize first the necessary resources, according to the service capabilities.

### Care Provision

Providers should be aware that telepsychiatry carries some additional challenges in regard to establishing rapport. This is due to the loss of nonverbal cues during the interaction, the lack of physical closeness, and, in cases where it is conducted *via* video conferencing, the artificiality of eye contact through a screen (16). Providers should demonstrate a high tone of professionalism in their verbal communication, maintaining an attitude of active, empathic, and non-judgmental listening. They should act as if they were, in fact, face-to-face with the caller, being mindful of their background and self-presentation, and trying to avoid multitasking and getting distracted during the conversation.

When contacted for the first time, providers should conduct an immediate assessment and intervention, tailored to each person’s needs to the best of the provider’s capabilities. **Table 3** shows a suggested guideline for conducting this initial assessment. After greeting the caller and introducing themselves, providers would set the frame of the consultation, assuring callers about its confidentiality, and obtain their informed consent. Providers will then ask for a name and contact information, the latter due to the possibility of having the call dropped out in the middle of a conversation; however, providers should be open to the possibility of callers not willing to share that information through telecommunication means. The initial assessment will then move to explore four areas: the caller’s current living conditions, the presence and quality of any psychological distress, COVID-19 diagnosis and misinformation about it, and the caller’s medical history. This assessment could also include the administration of screening scales, such as the Patient Health Questionnaire 9-item Depression scale (PHQ-9) and the 7-item Generalized Anxiety Disorder scale (GAD-7), two validated and widely used depression and anxiety measures (17, 18).

**Table 3:** Semi-structured outline of an initial assessment.

Good Morning/Afternoon/Night. Welcome to (name of hotline or department, if applicable). My name is ……. I am a mental healthcare provider/mental health specialist (specify) and I belong to [institution]. Could I get your name and your contact information, please? Hi [name], I need to ask you some questions to better determine how I can help you. Should I proceed?
**Explore current living conditions**
Presence or absence of social networks, support, resources, and challenges Explore loneliness and the individual’s subjective experience of physical distancing, presence and quality of social support in the house, whether the person is caring for people at home and if they are and feel supported in that care. Examples of questions: - Who lives with you at this moment? - How is your relationship with your family/people you are living with? - Do you have children or senior citizens living at home? - Are you caring for someone (ill or not) at home?
Employment and financial situation Type of work and working conditions, whether working or not, economic situation. Examples of questions: - What is your job? - Are you currently working? - Are you working on essential services? - Are you a healthcare professional? - Is anyone currently living with you a healthcare professional or working on essential services? - Are you currently facing financial issues due to the COVID-19 pandemic?
Disconnection from previous hobbies, leisure activities, and coping strategies Explore hobbies, presence or absence, and previously used coping strategies such as physical activities, eating outside, social gatherings. Examples of questions: - Did you use to go for walks or to the gym? - Did you use to care for your garden? Are you still doing it? - Are you still able to chat with your friends over the phone?
**Explore psychological distress and coping strategies**
Explore specific situations, thoughts, and emotions related to any perceived psychological distress. Examples of questions: - Are you currently experiencing any distressing emotion, sensation, or feeling? How long have you been feeling that way? How often? How strong is it? - Is there any specific situation worsening that feeling? - What do you normally do when you feel that way?
**Explore COVID-19 diagnosis and misinformation about COVID-19**
Explore whether the person or someone close to the person is a confirmed or probable case of COVID-19 and the measures adopted. Explore knowledge about the virus, transmission, symptoms of COVID-19, and individual and public health measures used to battle the pandemic. Examples of questions: - If you were tested, do you suspect you would have right now high chances to test positive for COVID-19? Why? - Where do you seek for information about the virus and COVID-19? - Have you been tested for COVID-19? Are you in isolation due to COVID-19 positivity? - Do you have someone at home in isolation due to COVID-19 positivity?
**Medical history**
Explore any previous or current psychiatric diagnosis, as well any comorbid physical condition. Examples of questions: - Do you have any diagnosis of a psychiatric or mental health condition, including substance and behavioral addictions? - Do you have any family members with a psychiatric diagnosis? - Do you have any general medical condition, such as diabetes, asthma, hypertension, other chronic or oncological diseases? - Do you use any substance (including alcohol and tobacco)? [Explore pattern of use] - Are you taking any medication or natural supplement? Are you following any psychological treatment?
**Scales**
Explore the presence or absence of psychiatric symptomatology *via* well established and validated scales, such as GAD-7 and PHQ-9.
**Note:** People matching the below conditions at the moment of the call should be further assessed by the mental health specialist or referred to an emergency service within the same call and as soon as possible: - while experiencing a psychiatric emergency (e.g. acute psychosis or suicidality), or - due to worsening psychological and/or psychiatric symptomatology, or - for being in a situation that places them or others at risk of harm, or - calling on behalf of someone in any of these situations. Providers will follow the department’s pre-established guidelines for referrals and all other necessary immediate actions, such as contacting emergency services.

As an outcome of this initial assessment, callers will then be matched to one of three potential lines of intervention (**Table 4**). Most people contacting the department will likely match either the first or the second-line interventions (**Table 5**). First-line interventions aim to provide trustworthy and appropriate information, reduce the distress associated with the pandemic and manage its emotional impact, and assist in the process of complying with public health measures of physical distancing. Providers who are not mental health specialists should be able to provide first-line interventions. Second-line interventions aim at providing the necessary support to people who are facing situations of particular distress or may be more susceptible to the mental health impact of the pandemic. Second-line interventions will require contact with a mental health specialist, whether during the same call or by referral to a scheduled one. Close monitoring *via* follow up calls and further communication should be scheduled with this population. People experiencing a psychiatric emergency or a situation that places them or others at risk of harm require third-line interventions, which include immediate contact with a mental health specialist or an emergency service.

**Table 4:** Target population and matching interventions.

First-line interventions: People with mild-intensity needs. People with no known psychiatric or physical condition showing signs of psychological distress due to uncertainty or misinformation, financial concerns, or physical distancing and self-isolation.
Second-line interventions: People with moderate-intensity needs. Health care workers and people providing essential services. People with a stable psychiatric or general medical condition or those caring for them, including people with chronic health conditions, neurodevelopmental disorders or intellectual disabilities, or older adults in need of constant home-based assistance. People with COVID-19 in forced self-isolation due to asymptomatic condition or mild flu-like symptoms, or people being treated for or recovering from COVID-19, as well as those caring for them, including healthcare workers or other professionals. Particular attention should be paid to those with comorbid mental health disorders.
Third-line interventions: People with high-intensity needs. People who present with worsening or uncontrolled psychological and/or psychiatric symptomatology. People grieving the loss of someone due to COVID-19. Psychiatric emergencies, including but not limited to suicide ideation, suicide attempt, and alcohol and/or other substance intoxication or severe withdrawal symptoms. People at risk of self-harm behaviors, harm to others, or harm from others, including victims of any type of violence.

**Table 5:** First, second, and third-line interventions.

**First-line:** First-line interventions include
Providing appropriate information about COVID-19 and public health measures. Recommending trusted sources, yet, recommending not to get overloaded or obsessed with information beyond what is needed to know in order to stay safe and avoid the spread of the infection.
Validating and normalizing the emotional response to the general situation, specific circumstances, or physical distancing and self-isolation. Explaining that worry, to a certain extent, is a normal coping mechanism.
Offering strategies to stay physically, mentally, and socially healthy, coping with the stress and boredom produced by physical distancing and self-isolation: Healthy daily routines, including eating and sleeping habits, and leisure activities. Physical exercise. Advice regarding how to improve social interactions with the people living with them (if applicable) and maintaining or enriching group and one-on-one social connections *via* phone calls, instant messaging, or video calls. Training and practicing relaxation and mindfulness techniques. For service users without previous experience in these techniques, training could be offered during the call or by referring to online resources and apps.
Providing information about any financial assistance available to the population in times of quarantine.
Scheduling a follow up call.
**Second-line:** Second-line interventions include (besides those previously mentioned in the first-line)
Providing strategies to cope with the fear of infection or spreading the virus to family, friends, and colleagues.
Providing additional advice for self-care to those caring for others (“caring for the carer”).
Emphasizing the need to continue with any prescribed psychiatric or general medical treatment or to continue to provide it to those under their care.
Note: Second-line interventions should always be assisted by a mental health specialist, whether in the same call or *via* a scheduled call to one, and follow up calls should always be scheduled with this population.
**Third-line:** Third-line interventions include
Contacting emergency services (police or an ambulance).
Referring callers to a specialized mental health care provider without losing contact with them.
Contacting a caller’s support person to assist or asking the caller to put one on the call.
Providing emotional support to the person calling on behalf of someone with high-intensity needs, while simultaneously contacting the police or ambulance, or referring the caller to a specialized mental health care provider.

All interventions should follow the criteria of appropriateness and evidence-based efficacy. It is highly recommended for providers to review and follow, according to the provider’s and service capabilities, the best practice guidelines provided by either local or international entities, e.g., those published by the American Psychiatric Association and the American Telemedicine Association (19), and other researchers (20). Finally, as much as with face-to-face consultations, cultural responsiveness is an essential component of telepsychiatry. All providers should be sensitive to the caller’s cultural identity and cultural conceptualisation of distress, as well as the impact of cultural features on the caller-provider relationship (DSM-5) (21). There is one extra component that needs to be taken into account, that is, the influence of the service user’s cultural background on the use of the provided service (22, 23). Providers should assess callers and communities’ acceptability of this service, adapt and respond to this assessment, and continuously monitor changes.

## Discussion

The rapid progression of COVID-19 and the non-pharmacological interventions adopted to reduce the spread of the virus have led to increasing difficulties in the provision of mental health care. As a result, telemedicine has become a pivotal tool in the task of ensuring the continuous provision of mental health care for the population. It is extremely important for mental health services around the globe to prepare and take action (24); the protocol here outlined can assist them with both.

The strength of this protocol lies in its practicality, clinical usefulness, and wide transferability, resulting from the diversity of the consensus group that developed it. Country representatives from a wide range of social, cultural, and economic contexts contributed to the development of this protocol. As such, it represents a valuable tool with a likely wide transferability across different regions and contexts. Nevertheless, it should be acknowledged that there is a potentially high degree of resource allocation needed to apply these recommendations, which may indeed limit its transferability to some contexts. Therefore, further studies are recommended to ensure a match between the here proposed protocol and country/context-specific conditions or to guide all necessary adjustments before it is implemented locally. Moreover, both these studies and/or the implementation of the present protocol in any mental health service should abide by specific local health regulations and the institutions’ ethics committees.

Still, the proposed protocol could help mental health providers to identify and address the mental health impact of physical distancing and misinformation during the COVID-19 pandemic, two key issues highlighted in the literature (25). Furthermore, mental health departments should also acknowledge the impact of physical distancing and provide adequate mental health support (26). This support may prove a valuable resource in assisting people to comply with prescribed physical distancing measures, and thus with the battle against the pandemic (27, 28). The WHO has called people to resort only to official sources when seeking information about COVID-19 (29). Mental health departments should support this call and combat misinformation, but they should also provide people with strategies to avoid a hyperconsumption of information (30, 31). These are all points addressed in the recommended protocol and should be acknowledged when developing local adaptations.

The literature also suggests that it may prove beneficial to develop targeted telepsychiatry interventions for different populations during the pandemic (32). Particular attention should be paid to older adults, children, and those caring for them (33–35). Similarly, healthcare professionals require special consideration (30, 36, 37). The proposed protocol would allow mental health departments to identify and support these and other particularly vulnerable populations, such as those with a pre-existing or emerging mental health disorder (5, 38) and those in situations of domestic or intrafamilial violence, which may dramatically increase during the quarantine (39). It is highly recommended for local adaptions of this protocol also to identify and cater for different populations within each specific context in case these were not contemplated in the present protocol.

As suggested by other authors (6, 26, 40), telemedicine services should be formally provided as a crucial component of the public health response to the ongoing COVID-19 pandemic. The adoption and expansion of telepsychiatry in mental health care would simultaneously improve access to this care and decongest those mental health care services already working at capacity. The proposed protocol can support mental health departments to provide care in non-urgent situations that do not necessarily require a face-to-face interaction, minimizing the risk of contagion between members of the population and the health care workforce. It can also help with redirecting and maximizing the use of available resources, including specialized mental health care professionals. But further research is needed on its use and applicability to local healthcare systems, services, and resources during the pandemic.

The mental health impact of the COVID-19 pandemic has forced mental health services around the world to adapt. The adoption of this protocol can complement existing guidelines during the pandemic in those contexts where telepsychiatry was well established already, but most importantly, it can also provide a starting point to those where telepsychiatry has played a marginal role until now. These are particularly difficult moments in time. However, they also offer the opportunity to advance the way in which mental health services worldwide support the population, and this protocol also highlights the importance of acknowledging and harvesting the knowledge and expertise of early career psychiatrists around the globe in that task.

## Data Availability Statement

The original contributions presented in the study are included in the article, further inquiries can be directed to the corresponding author.

## Author Contributions

RRam developed the trigger questions and topics and wrote the first draft of recommendations and article with the support from LO. All authors contributed to the article and approved the submitted version.

## Conflict of Interest

The authors declare that the research was conducted in the absence of any commercial or financial relationships that could be construed as a potential conflict of interest.
